# Self-care practices and associated factors among hypertension patients in public hospitals in Harari regional state and Dire Dawa City administration, Eastern Ethiopia: A multi-center cross-sectional study

**DOI:** 10.3389/fpubh.2022.911593

**Published:** 2022-08-05

**Authors:** Lemesa Abdisa, Bikila Balis, Kasiye Shiferaw, Adera Debella, Habtamu Bekele, Sagni Girma, Ayalnesh Mechal, Eldana Amare, Temesgen Kechine, Kajela Tari, Kabtamu Nigussie, Nega Assefa, Shiferaw Letta

**Affiliations:** ^1^School of Nursing and Midwifery, College of Health and Medical Science, Haramaya University, Harar, Ethiopia; ^2^Department of Nursing, College of Medicine and Health Science, Wachemo University, Hosaena, Ethiopia; ^3^Department of Nursing and Midwifery, College of Medical and Health Science, Dilla University, Dilla, Ethiopia

**Keywords:** self-care, hypertensive, associated factors, Eastern, Ethiopia

## Abstract

**Introduction:**

Hypertension is a silent killer that causes serious health issues in all parts of the world. Hypertension is a risk factor for cardiovascular disease, stroke, and kidney disease. Self-care practices have been identified as an important component of hypertension management. Despite the government's commitment and the interventions of various stakeholders, the burden of hypertension and its sequel remain unabated. A recent study showed that hypertension self-care practices play a vital role in controlling and managing high blood pressure, even though there is poor self-practice among hypertensive patients in Ethiopia. Therefore, this study assessed the level of self-care practices and associated factors among hypertension patients in public hospitals in Harari regional state and Dire Dawa City Administration, Eastern Ethiopia.

**Methods:**

Hospital-based cross-sectional study was conducted from June 15 to July 15/2021 among 415 adult hypertensive patients on follow-up. The participants were selected using systematic sampling. Hypertension Self-Care Activity Level Effects (H-SCALE) was used to collect data through face-to-face interviews. The SPSS version 24 was used for analysis. Logistic regression analyses were done to determine the association between the outcome and independent variables. For multivariate logistic regression models, variables having a *P* < 0.25 during bivariate analysis were candidates. The strength of the association was estimated using AOR and 95% CI. The level of statistical significance was declared at a *p* < 0.05.

**Results:**

This study revealed that 52% (95% CI, 48.2–58%) had good level of self-care practices. Formal education (AOR = 3.45, 95% CI: 2.1–4.85), good knowledge about hypertension (AOR = 1.5, 95% CI: 1.17–2.1) 1.5, abstain from chewing khat (AOR = 2.01, 95% CI: 1.44–3.94), strong social support (AOR= 1.9, 95% CI: 1.16–3.1), and absence of depression (AOR = 2.03, 95% CI: 1.43–3.92) were statistically associated with a good level of self-care practices.

**Conclusions:**

This study pointed out that about half of the participants had a good level of self-care practices. Formal education, good knowledge about hypertension, abstaining from khat chewing, good social support, and absence of depression showed associations with a good level of self-care practices. Therefore, public health interventions on hypertension self-care practices, and strengthening non-communicable diseases control programs are vital. Moreover, the provision of targeted education to patients can improve disease knowledge and self-care practices.

## Introduction

According to a global report on high blood pressure, about 1.39 billion people had hypertension in 2010 (1). The trend of hypertension is shifting from developed countries to developing countries, where there is little knowledge of hypertension and its management (1, 2); an estimated 349 million in developed countries and 1.04 billion in developing countries suffer from hypertension (1).

In sub-Saharan Africa (SSA), high blood pressure projections will be between 125.5 and 162.8 million by 2025 (3, 4). Even though no countrywide study was conducted in Ethiopia, the results of a systematic review and meta-analysis revealed that hypertension was prevalent in 21.81 percent of the population (5). Despite the availability of hypertension treatment options, the majority of hypertensive patients are living with uncontrolled hypertension (6).

Hypertension is a risk factor for health failure, stroke, and Kidney diseases (7). Uncontrolled hypertension causes 10.4 million deaths per year globally (8). Increasing systolic blood pressure (SBP) by 20 mmHg and diastolic blood pressure (DBP) by 10 mmHg above normal ranges the risk of cardiovascular diseases, strokes, and kidney diseases are doubled (9, 10). The report of the World Health Organization (WHO) showed that uncontrolled hypertension cause 9.4 million complications and 17 million deaths due to cardiovascular diseases (CVD) globally (11).

Self-care activities are recommended by the World Health Organization (WHO) to improve the efficacy of antihypertensive drugs (12). Compliance with self-care practices helps hypertensive patients to control their blood pressure; prevent and reduce complications; and subsequent morbidities, disabilities, and death (13).

Hypertension self-care practices are a dynamic and active process that necessitates knowledge, attitude, discipline, determination, commitment, self-regulation, empowerment, and self-efficacy to manage diseases and achieve healthy living (14). It contains taking medications, consumption of a low-salt diet, moderating alcohol drinking, physical exercises, weight management, not smoking, blood pressure monitoring, and reducing stress (15, 16). Previous showed that sex, age, occupational status, time since diagnosis, comorbidities, knowledge about the disease, self-efficacy, social support, smoking, and khat chewing are associated with components of self-care practice of hypertension (17–20).

Though there have been few documented studies on self-care practices among hypertensive patients in Ethiopia, the roles of depression and anxiety were not studied. Moreover, to the knowledge of the researchers, there is paucity of evidence about self-care practices and associated factors in the study area. Therefore, this study assessed the level of self-care practices and associated factors among hypertension patients in public hospitals in Harari regional state and Dire Dawa City Administration, Eastern Ethiopia.

## Materials and Methods

### Study design and area

A hospital-based cross-sectional study was conducted in the Harari region state and Dire Dawa City Administration, Eastern Ethiopia from June 15 to July 15, 2021. Harari regional state is one of the eleven states in Ethiopia. Harar is the capital city of the Harari region found 526 km distance from the southeast of Addis Ababa. There are two public Hospitals found in Harar regional state; Hiwot Fana Compressive Specialized University Hospital (HCFSUH) and Jugal hospitals (JH). Based on the 2007 Central Statistical Agency population census, the total population of the town was projected to be 259,260, of those 130,097 are females in 2021.

Dire Dawa City Administration is one of the two federal city Administrations in Ethiopia. It is found in the Eastern part of Ethiopia at a distance of 515 km away from Addis Ababa. There are two public Hospitals found in Dire Dawa City Administration; Dilchora Referal Hospital (DRH) and Sabian Hospital (SGH). According to the 2007 Central Statistical Agency population census, the total population of the Dire Dawa has an estimated population of 599,651, of whom 301,496 are females in 2021.

### Study participants and eligibility criteria

All hypertensive patients on follow-up at the selected public hospitals during the study period and fulfilled the inclusion criteria were the study population. Patients aged ≥18 years and taking antihypertensive drugs for ≥6 months were included in the study. Patients who had cognitive impairment and who were severely ill were excluded from the study since they cannot provide valid information.

### Sampling and sampling procedures

Sample size was calculated by using single population proportion formula with the assumptions of: Zα/2 = 1.96, 95% confidence level, 5% margin of error, the prevalence of good self-care practices (*p* = 0.49) (18), with 10% for non-response rate, and yields a total sample size of 422. For the previous 3 months, clients' flow to each hospital for hypertension follow-up was reviewed from the registration book to estimate the expected number of patients that will come for the follow-up in 1 month (study period). Based on this the expected number of hypertensive patients who will come for the next month was 978. Then calculated sample size (422) was allocated to each hospital based on their respective expected number of patients who come in 1 month period. Finally, study participants were selected by using systematic random sampling of every *K*-value (2). K = N/n, 978/422 with k = 2.3 ≈ 2. The first patient were selected by the lottery method.

### Data collection tool and procedures

A pretested and validated interviewer-administered questionnaire was used for data collection. The questionnaire contains five parts: part I: socioeconomic characteristics; part II hypertension Knowledge-Level Scale (HK-LS) (21). The internal consistency was checked by using Cronbach's alpha and it was 0.81. Part III: Hypertension Self-Care Activity Level Effects (H-SCALE) (22). In this study, internal consistency was checked by Cronbach's alpha which was (α = 0.85). The Cronbach's alpha of sub-domains; medication adherence, low salt, physical activity adherence, weight management, and alcohol use were 0.94, 0.74, 0.81, 0.93, and 0.92, respectively. Part IV clinical-related characteristics, and part V psychosocial related factors like anxiety, depression, and social support. The internal consistency of the Generalized Anxiety Disorder 7-item (GAD-7) scale was 0.90 (23); whereas the internal consistency of the Patient Health Questionnaire-9 (PHQ-9) was 0.95 (24), and social support was assessed by Oslo Social Support Scale (OSSS-3), and its internal consistency was 0.86 (25). Data were collected by eight trained BSc nurses and supervised by four MSc nurses.

### Study variables and measurements

Dependent variable: Hypertension self-care practice. Independent variables: Socio-demographic variables: sex, age marital status, religion, educational level, place of residence, and monthly income. Hypertension knowledge. Clinically related variables: body mass index, duration of diagnosis, frequency of follow-up, follow-up miss, comorbidity. Psychosocial related factors: anxiety, depression, and social support.

Hypertension self-care practice was measured using Hypertension Self-Care Activity Level Effects (H-SCALE) which contains six domains (medication adherence, weight management, physical exercises, smoking, alcohol intake, and low salt diet) (26). The patients who responds to four or >6 domains of H-SCALE were considered to have “good self-care practices” unless “poor self-care practices.” Medication adherence was measured by three items containing the number of days in the last 7 days. Responses were summed (range: 0–21). Patients who scored 21 were considered adherent to antihypertensive medication (26). Weigh management was measured by ten Likert-type items rated from 1 (strongly disagree) to 5 (strongly agree). The responses summed (10-50). Patients scored ≥40 were considered adherent to weight management (26). A low salt diet was measured by 12 items. The mean was calculated. A patient who scored ≥6 (indicating participants followed low salt diet practice on 6 out of 7 days) was considered adherent to a low-salt diet (26). Physical activity was assessed by 2 items. Responses were scored (range: 0–14). The patients who scored ≥8 were considered physically activate (26). Smoking was assessed by one whether the patients smoked in the last week. Patients who had not smoked in the last seven days were considered as a non-smoker (26). Alcohol intake was assessed by three items. Patients who did not drink alcohol at all were considered abstainers (26).

Hypertension Knowledge was measured by HK-LS which contains 22 item questions. Nine of these items on the questionnaire were negatively phrased. Before the analysis, these were reversely scored. The total sum of the scores of the knowledge items gives a score ranging from 0 to 22. The mean was calculated. Respondents who scored equal to mean and above were considered as having “good knowledge about hypertension” unless poor knowledge.

Anxiety was assessed by the Generalized Anxiety Disorder 7-item (GAD-7) scale. Seven Likert-type scales were from 0 (not at all) to 3 (nearly every day); which gives a score ranging from 0 to 21. In the current study, patients with a score of ≥ 10 had anxiety (27).

Depression was screened using the patient health questionnaire-9 (PHQ-9). The nine Likert-type scales are scored from 0 (not at all) to 3 (nearly every day); with a score ranging from 0 to 27. In the current study, patients with a score of ≥ 10 had depression (23).

The level of social support was measured using Oslo social support scale (OSSS-3) which contains three items. The first item is rated on a four-point Likert scale ranging from 1 to 4. The second and the third items are rated on a five-point Likert scale ranging from 1 to 5. The sum score ranges from 3 to 14. The ranges from 12 to 14 to OSSS-3 was strong social support, 9–11 was considered moderate social support, and 3–8 was poor social support (28).

### Data quality control

The questionnaire was initially prepared in English and then translated into the local languages Language experts then translated it back into English to ensure consistency. The data collectors and field supervisors were received training on the data collection tool and procedures. Before the actual study, the pretest was conducted among 5% (twenty-one) of the total sample at Haramaya General Hospital. During pretesting, the questionnaire was checked for its clarity, simplicity, understandability, consistency, and coherency. During the data collection period, close supervision was done by the supervisor.

### Data processing and analysis

The data were entered into Epi data version 3.1 and exported to Statistical Package for the Social Sciences (SPSS) version 20 for statistical analysis. Descriptive analyses were presented using frequency, percent, mean, and standard deviation. Bivariable and multivariable binary logistic regression analyses were used to see the association between independent variables and the outcome. Predictors' variables with a *p* < 0.25 in bivariable were included in the multivariable model. The association between outcome variables and predictors was presented by the adjusted odds ratio (AORs) with a 95% confidence interval. The Hosmer-Lemeshow statistics indicate a good fit at a *p* < 0.05 or greater.

### Ethical consideration

Ethical clearance was obtained from the Institutional Health Research Ethics Review Committee (IHRERC) of Haramaya University College of Health and Medical Sciences. A formal letter of permission and support was provided to all the four public hospitals in which the study was conducted. Informed, voluntary, written, and signed consent was obtained from the heads of the respective hospitals. Participants were informed about the aim of the study and the advantage of the study; confidentiality, there was no risk of being participants, and they have full right to halt in the middle of the interview. Oral and written informed consent was taken from each participant before data collection began. Confidentiality was maintained at all levels of the study through anonymous data collection. During data collection, the COVID-19 prevention protocol was kept.

## Results

### Sociodemographic characteristics

A total of 422 participants were involved in this study, with a response rate of 100%. The age of the participants ranged from 24 to 79 years with a mean age of 45 (SD = ±12). About 220 (53%) were males and 264 (62.6%) of the patients were married and 67.3% of them were urban residents. One hundred seventy-four (41.2%) were Muslim religion followers and almost half, 200 (47.4%) of the respondents attended college and above. Regarding Khat chewing among the study participants 148 (35.1%) of them chewed khat in the last 30 days. Level of social support 167 (39.6%) of the participants had strong social support, 117 (27.7%) participants had moderate social support and 138 (32.7%) of them had poor social support (Table 1).

**Table 1:** Sociodemographic characteristics of hypertensive patients on follow-up at public Hospitals in the Harari regional state and Dire Dawa City administration, Eastern Ethiopia, 2022 (*n* = 422).

**Variable**	**Frequency**	**Percent**
	**(*n* = 422)**	**(%)**
**Sex**
Male	224	53
Female	198	47
**Age (years)**
<50	208	49.3
≥50	214	50.7
**Marital status**
Single	28	6.6
Married	264	62.6
Divorced	47	11.1
Widowed	83	19.7
**Religion**
Muslim	174	41.2
Orthodox	156	37
Protestant	67	15.8
Others*	25	6
**Educational status**
No formal education	68	16.1
Primary education	56	13.3
Secondary education	98	23.2
College and above	200	47.4
**Place of residence**
Urban	284	67.3
Rural	138	32.7
**Monthly income (ETB)**
≤ 500	47	11.1
501–2,000	99	23.5
>2,000	276	65.4
**Khat chewing**
Yes	148	35.1
No	274	64.9
**Level of social support**
Strong social support	167	39.6
Moderate social support	117	27.7
Poor social support	138	32.7

* Others: Catholic, Waqefata.

### Clinical related characteristics

The majority of participants, 255 (60.4%), had normal body mass index followed by an overweight 80 (19%). Regarding the duration of diagnosis with HTN, almost half of 215 (51.8%) of the patients, were diagnosed <5 years ago. Almost half, 216 (52%), had a 3-month follow-up appointment, and about 208 (52%), had a history of missed follow-ups. About 182 (43.1%), of the patients, had other comorbidity/s, and the majority, 111 (60%) had diabetes mellitus. Among the respondents, 94 (22.27%) had anxiety and 85 (20.14%) had depression (Table 2).

**Table 2:** Patient and clinical characteristics of hypertensive patients on follow-up at public Hospitals in the Harari regional state and Dire Dawa City administration, Eastern Ethiopia, 2022 (n = 422).

**Variable**	**Frequency**	**Percent**
	**(*n* = 422)**	**(%)**
**Body mass index (kg/m** ^**2**^ **)**		
18.5–24.9	255	60.4
25–29.9	80	19
≥30	24	5.6
<18.5	63	15
**Duration of hypertension diagnosis (years)**		
<5	215	50.9
5–10	135	32
≥10	72	17.1
**Number of medication**		
Monotherapy	112	26.5
Dual therapy	196	46.5
≥Triple therapy	114	27
**Follow-up miss**		
Yes	215	50.9
No	207	49.1
**Comorbidity**		
Yes	182	43.1
No	240	56.9
**Types of comorbidities (*****n*** **=** **182)**		
Diabetes mellitus	111	61
Chronic kidney disease	23	12.6
Myocardial infarction	33	18.1
Stroke	26	14.3
Others*	29	15.9
**Knowledge of patient about hypertension and self-care**		
Good	253	60
Poor	169	40
**Anxiety**		
Yes	94	22.3
No	328	77.7
**Depression**		
Yes	85	20.1
No	337	79.9

Others: ^*^Heart failure, hyperlipidemia, and ischemic heart disease.

### Hypertension self-care practices

**Figure 1:**
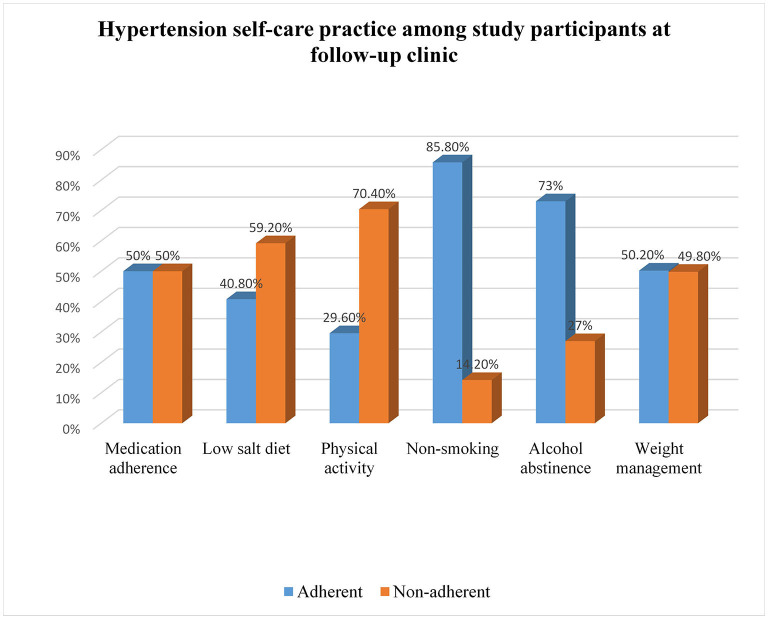
Hypertension self-care practices of hypertensive patients on follow-up at public Hospitals in the Harari regional state and Dire Dawa City administration, Eastern Ethiopia, 2022 (*n* = 422).

About two hundred twenty 52% (95% CI, 48.2–58%) of the patients had good hypertension self-care practices (SCPs). The mean score of the patient's knowledge about hypertension was 13.02 + 3.72 with ranges of 2 to 21. The majority, 253 (60%) had good knowledge about hypertension while the rest had poor knowledge about hypertension 50, 40.8, 29.6, 85.3, 73, 50.2% were adherent to antihypertensive medication, low-salt diet, physical activity, smoking abstains, alcohol abstainer, and weight management (Figure 1).

### Bivariable and multivariable analysis

In bivariable analysis, sex, age, educational level, place of residence, number of antihypertensives, Knowledge about hypertension and treatment, chewing khat, social support, and depression were significantly associated with a good self-care practice at a *p* < 0.25. In the multivariable logistic regression, educational level, knowledge about hypertension and treatment, chewing khat, social support, and depression were independent predictors of self-care practices at a *p* < 0.05 (Table 3).

**Table 3:** Factors associated with self-care practices of hypertensive patients on follow-up at public Hospitals in the Harari regional state and Dire Dawa City administration, Eastern Ethiopia, 2022 (*n* = 422).

**Variables**	**Hypertension self-care practices**		**COR (95% CI)**	**AOR (95% CI)**
	**Good (%)**	**Poor (%)**		
**Sex**
Female	120 (60.6)	78 (39.4)	1.90 (1.12–2.45)	1.64 (0.96–2.26)
Male	100 (44.6)	124 (55.4)	1	1
**Age**
<50	114 (54.8)	94 (45.2)	1.23 (1.02–1.98)	1.01 (0.67–1.78)
≥50	106 (49.5)	108 (50.5)	1	1
**Educational level**
Formal education	201 (56.8%)	153 (43.2%)	3.39 (1.88–6.98)	2.45 (1.18–4.85)**
No formal education	19 (27.9)	49 (72.1)	1	1
**Place of residence**
Urban	155 (54.6)	129 (45.4)	1.35 (1.12–2.13)	1.09 (0.68–1.76)
Rural	65 (46.7)	73 (53.3)	1	
**Number medication**
≤ Two	174 (56.5)	134 (43.5)	1.92 (1.222–3.56)	1.40 (0.84–2.91)
≥Three	46 (40.4)	68 (59.6)	1	1
**Knowledge about hypertension**
Good	170 (67.2)	83 (32.8)	4.87 (2.33–7.77)	3.5 (2.17–5.12)**
Poor	50 (29.6)	119 (70.4)	1	1
**Khat chewing**
No	173 (63.1)	101 (36.9)	3.68 (1.55–3.88)	2.01 (1.44–3.94)**
Yes	47 (31.8)	101 (68.2)	1	1
**Level of social support**
Strong	101 (63.1)	59 (36.9)	2.58 (1.36–3.44)	1.92 (1.16–3.11)*
Moderate	64 (51.6)	60 (48.4%)	1.58 (0.73–1.97)	1.07 (0.64–1.8)
Poor	55 (39.9)	83 (60.1)	1	1
**Depression**
No	191 (56.7)	146 (43.3)	2.53 (1.41–4.70)	1.63 (1.23–3.92)*
Yes	29 (34.1)	56 (65.9)	1	1

* P < 0.05 and ^**^p < 0.001; Hosmer and Lemeshow Test = 0.98.

1 = Reference.

Accordingly, participants who received formal education were 2.45 times more likely to practice good self-care than those who did not receive a formal education (AOR = 2.45, 95% CI: 1.18–4.85). The odds of having good self-care among participants with good hypertension knowledge were 3.5 times higher than those with poor knowledge (AOR = 3.5, 95% CI: 2.17–5.12). Patients who did not chew khat were twice as likely as their counterparts to practice good hypertension self-care (AOR = 2.01, 95% CI: 1.44–3.94). Those who received strong social support were 1.9 times more likely to practice good hypertension self-care than those who received poor social support (AOR = 1.92, 95% CI: 1.16–3.11). Patients without depression were 1.63 more likely as compared to patients with depression to have good self-care practices (AOR = 1.63 95% CI: 1.23–3.92).

## Discussion

This study was assessed self-care practices and associated factors among hypertensive patients in public hospitals, in Eastern Ethiopia. This study revealed that 52% (95% CI, 48.2–58%) of hypertensive patients had good SCPs. According to this study, ~1 out of every two hypertensive patients have good self-care practices. Those who have a formal education, a good knowledge about hypertension, those did not chew khat, having strong social support, and those did not have depression were significantly associated with self-care practices.

According to findings from this study, 52% (95% CI, 48.2–58%) of hypertensive patients had good SCPs. A similar finding was reported in studies conducted in South India (52.4%) (29), Debre Tabor, Ethiopia (54.1%) (17), Dessie town (49%) (18), and Addis Ababa (51%) (30). The finding was higher than the studies conducted in West Bengal (37.1%) (31), Harar (29.9%) (32), Debre Berhan 24% (17), Mekele (20.3%) (33), and Addis Ababa (39.5%) (34). This could be explained by sample size differences, socio-cultural variations, and levels of knowledge about hypertension and management. On contrary, this finding was relatively lower than studies conducted in Nekemte (68.92%) (35), Gondar (59.4%) (36), Harar (62.1%) (19), and Saudi Arabia (74.4%) (20). This disparity could be attributed to assessment tools as well as socioeconomic status. For instance, a study in Harar adapted from WHO STEP surveillance, but in this study, we used H-SCALE to assess self-care practices.

Our study showed that having formal education was 3.45 more likely to have hypertension self-care practices than their counterparts. This is in line with studies done in Dessie town (18), Harar (32), Malaysia (37), and India (31). The possible explanation might be educated people can understand by reading things that are useful for hypertension self-care and they shall understand recommended lifestyle modifications to prevent hypertension and its complication (18, 32). This implies the need to design an educational intervention convenient for those who hadn't formal education.

Good knowledge about hypertension and treatment was 1.5 times more associated with good self-care practices. This finding consistent with is similar to studies done in Debre Tabor (17), Harar (32), Mekele (33), Addis Ababa (38), and Saudi Arabia (20). The possible justification is because knowledgeable patients know about the disease, treatment, complications, and how it can be controlled and managed. Additionally, patients having good knowledge about hypertension and its treatment give more emphasis to self-care practices.

Our study showed that patients who did not chew khat were two times more likely to have good hypertension SCPs than khat chewers. This finding was consistent with studies done in Harar, Ethiopia (19, 32). It might be due to individuals who chew the khat being more likely to engage in alcohol drinking & smoking as reported in other studies (19, 32). Another possible justification is due to the effect of khat on psychoactive which results in forgetting recommended self-care practices (39).

The odds of having good self-care practices (SCPs) were 1.9 higher than those with strong social support as compared to those who had poor social support. This is in line with studies conducted in Harar (32), Dessie town (18), Debre Tabor (17), and China (40). This might be due to the existence of the family or relative support increasing adherence to components of self-care practices. The WHO self-care practices guideline supports the presence of good social support for coping with chronic diseases like hypertension (41). Prospective studies are also needed to determine the effects of social support on hypertension self-care practice.

The hypertensive without depression were two more likely to have hypertension self-care practices (SCPs) than those who had depression. This finding is similar in line with the study reported in Korea (42). The possible justification might be a cognitive effect of depression which causes difficulty in concentrating and forgetfulness of recommended SCPs. This implies that those patients who have depression need special consideration.

### Limitations of the study

Since this study is a multicenter study it is a better representation of the study participants and generalizability of the result. Also in our study, we try to assess independent factors of depression and anxiety effects of hypertension self-care. This study has also some limitations, since data was collected by a self-report the patients may not memorize which leads to recall bias and may affect the results of this study.

## Conclusion

This study reported that more than half of the respondents have good hypertension self-care practices. Formal education, good knowledge about hypertension, abstaining from khat chewing, good social support, and no depression was positively associated with self-care practices. The policymakers should consider these factors in specific public health interventions on hypertension self-care practices, as well as in strengthening the current non-communicable diseases control programs. To make this happen, public health facilities should strengthen efforts to provide targeted education to patients and family members on all components of self-care practice. In addition, patients with depression need special consideration in hypertension self-care management.

## Data availability statement

Data can be made available on request to the corresponding authors.

## Ethics statement

The studies that included human participants were reviewed and approved by Haramaya University, Institutional Health, and Research Ethics Review Committee of the College of Health and Medical Sciences. The participants were given their written informed consent to participate in this study. Informed, voluntary, written, and signed consent was obtained from each study participant after explanations about the aims, objectives, benefits, and harms of the study were provided. The person's information was entirely confidential. During data collection, the COVID-19 prevention protocol was kept.

## Author contributions

All authors made essential contributions in designing, analyzing, interpreting, writing manuscripts, read and approve the final draft of the manuscript, and agreed on the final manuscript for publication.

## Funding

Haramaya University has funded the data collection of this study. The funders had no role in the design of the study, analysis, interpretation, and publishing of the manuscript.

## Conflict of interest

The authors declare that the research was conducted in the absence of any commercial or financial relationships that could be construed as a potential conflict of interest.

## Publisher's note

All claims expressed in this article are solely those of the authors and do not necessarily represent those of their affiliated organizations, or those of the publisher, the editors and the reviewers. Any product that may be evaluated in this article, or claim that may be made by its manufacturer, is not guaranteed or endorsed by the publisher.

## Abbreviations

AOR: Adjusted Odd Ratio
BP: Blood Pressure
CI: Confidence Interval
COR: Crude Odd Ration
CVD: Cardiovascular Diseases
GAD-7: Generalized Anxiety Disorder 7-item
H-SCALE: Hypertension self-care Activity Level Effects
HK-LS: Hypertension Knowledge-Level Scale
LMICs: Low and Middle-Income Countries
NCD: Non-communicable Diseases
OSSS-3: Oslo social support scale
PHQ-9: Patient Health Questionnaire-9 items
SCPs: Self-care Practices
SSA: Sub-Saharan Africa
WHO: World Health Organization.
